# Omalizumab in the treatment of severe allergic (IgE-mediated) asthma: an update on recent developments

**DOI:** 10.1186/2045-7022-3-S1-O12

**Published:** 2013-05-03

**Authors:** Marc Humbert, Janice Canvin, Tamara Kiechle, Phil Lowe, Veit Erpenbeck

**Affiliations:** 1 Hôpital Bicêtre, Hôpitaux Universitaires Paris-Sud, France; 2 Novartis Pharmaceuticals UK Limited, Clinical Development, UK; 3 Novartis Pharma AG, Medical Affairs, Switzerland; 4 Novartis Pharma AG, Modelling and Simulation, Switzerland; 5 Novartis Pharma AG, Translational Medicine, Switzerland

## 

Omalizumab (OMA), a humanized anti-immunoglobulin E (IgE) monoclonal antibody was approved in the EU in 2005 as an add-on therapy for patients with severe persistent allergic asthma (AA). We present an overview of recent developments in the use of OMA for this indication.

Approval for pediatric use: followed the completion of two clinical trials in children 6 to <12 years of age with moderate-to-severe AA, who were either well- or inadequately controlled with inhaled corticosteroids (CS). Pooled analysis of these data demonstrated the efficacy and safety of OMA and led to approval in the EU for use in this population.

## Expansion of the dosing table (DT)

The initial DT applied to patients with IgE levels between 30–700 IU/ml and body weight up to 150 kg, with a maximum dose of 375 mg per administration. The DT was expanded to enable treatment of patients with levels up to 1,500 IU/ml, with a maximal dose of 600 mg per administration. Subsequent pharmacokinetic (PK) and pharmacodynamic (PD) modelling and simulation predicted that some doses could be doubled and given less frequently, while maintaining efficient suppression of free IgE without compromising safety. The DT was further revised and approved in the EU in 2012, simplifying the dosing schedule in a subset of patients (225–300 mg every 2 weeks) to receive treatment at double the dose every 4 weeks. Development of a pre-filled syringe: A more concentrated liquid formulation may eliminate a potential source of error during dosing and simplify drug administration. A PK/PD study demonstrated bioequivalence and a similar safety and tolerability profile between the liquid formulation and the lyophilized powder.

## OMA safety update

A pooled analysis of 32 trials showed no association between treatment and risk of malignancy (rate ratio 0.93 [95% CI, 0.39–2.27]). In an interim analysis of an observational study (N=8,023), the malignancy incidence was similar in both cohorts (+/-OMA). OMA ‘real-life’ 2-year registry (N=943): Efficacy results demonstrated an increase in the number of patients free from clinically significant exacerbations following OMA treatment (6.8% at baseline to 67.3% at 24 months). A reduction in maintenance oral CS use to 14.2% after 24 months compared with 28.6% at baseline and, improvements in asthma control and quality of life were also observed. Such advances in the use of OMA may benefit a wider range of patients with severe AA and address unmet needs in asthma treatment.

